# Isolation of a strong *Arabidopsis *guard cell promoter and its potential as a research tool

**DOI:** 10.1186/1746-4811-4-6

**Published:** 2008-02-19

**Authors:** Yingzhen Yang, Alex Costa, Nathalie Leonhardt, Robert S Siegel, Julian I Schroeder

**Affiliations:** 1 Cell and Developmental Biology Section, Division of Biological Sciences, University of California, San Diego, 9500 Gilman Drive, La Jolla, CA, 92093-0116, USA; 2 Department of Biology, University of Padua, Via U. Bassi 58/B, I-35131, Padova, Italy; 3 CEA Cadarache, DSV, UMR 6191 CEA-CNRS, DEVM, LEMS and LEMP, St Paul les Durance Cedex, France

## Abstract

**Background:**

A common limitation in guard cell signaling research is that it is difficult to obtain consistent high expression of transgenes of interest in *Arabidopsis *guard cells using known guard cell promoters or the constitutive 35S cauliflower mosaic virus promoter. An additional drawback of the 35S promoter is that ectopically expressing a gene throughout the organism could cause pleiotropic effects. To improve available methods for targeted gene expression in guard cells, we isolated strong guard cell promoter candidates based on new guard cell-specific microarray analyses of 23,000 genes that are made available together with this report.

**Results:**

A promoter, *pGC1*(At1g22690), drove strong and relatively specific reporter gene expression in guard cells including GUS (beta-glucuronidase) and yellow cameleon YC3.60 (GFP-based calcium FRET reporter). Reporter gene expression was weaker in immature guard cells. The expression of YC3.60 was sufficiently strong to image intracellular Ca^2+ ^dynamics in guard cells of intact plants and resolved spontaneous calcium transients in guard cells. The *GC1 *promoter also mediated strong reporter expression in clustered stomata in the stomatal development mutant *too-many-mouths *(*tmm*). Furthermore, the same promoter::reporter constructs also drove guard cell specific reporter expression in tobacco, illustrating the potential of this promoter as a method for high level expression in guard cells. A serial deletion of the promoter defined a guard cell expression promoter region. In addition, anti-sense repression using *pGC1 *was powerful for reducing specific GFP gene expression in guard cells while expression in leaf epidermal cells was not repressed, demonstrating strong cell-type preferential gene repression.

**Conclusion:**

The *pGC1 *promoter described here drives strong reporter expression in guard cells of *Arabidopsis *and tobacco plants. It provides a potent research tool for targeted guard cell expression or gene silencing. It is also applicable to reduce specific gene expression in guard cells, providing a method for circumvention of limitations arising from genetic redundancy and lethality. These advances could be very useful for manipulating signaling pathways in guard cells and modifying plant performance under stress conditions. In addition, new guard cell and mesophyll cell-specific 23,000 gene microarray data are made publicly available here.

## Background

Stomata are located on the leaf surface and are the main conduit for water transpiration and CO_2 _influx into leaves. The stomatal aperture is regulated by multiple physiological factors such as light, CO_2_, and plant hormones including abscisic acid (ABA) [1-5]. These stimuli regulate the stomatal aperture by affecting the cellular activities of the two adjacent guard cells, which form the stomata.

Many genes are important for guard cell function as demonstrated by forward genetic screens and reverse genetic functional analyses. To improve plant performance under stress conditions, manipulating gene function specifically in guard cells offers advantages over manipulation at the whole plant level. For example, a dominant mutation or a knock out mutation in an essential gene at the whole plant level might be lethal. This problem could be avoided by expressing the mutated gene or silencing the specific gene in guard cells only. Secondary messengers, such as calcium, reactive oxygen species, inositol phosphates, and sphingolipids, have been shown to play a critical role in guard cell signaling [6-11]. Molecular reporters for some of these secondary messengers have been developed and used in mammalian cell biology, such as yellow cameleon (YC) for calcium [12] and Hyper for H_2_O_2 _[13]. Several calcium reporters have been used for studies in plant biology, including indo-1, fura-2, aequorin, and yellow cameleon [14-19]. Single cell imaging of second messengers in intact plants could provide an approach to analyze second messengers within the leaf and plant context. Intact plant imaging of single cells requires specific reporter gene expression in target cells with low background in the surrounding cells.

The widely used constitutive 35S cauliflower mosaic virus promoter drives expression of an interested gene in most parts of the plant [20]. The 35S promoter can also drive gene expression in guard cells [15,21-23]. One copy of the 35S promoter, however, often drives weak expression in guard cells while two tandem 35S promoters provides approximately two-fold higher expression [21,22]. In addition, gene expression driven by the 35S promoter is not always uniform in guard cells even in the same leaf. Furthermore, gene expression in many different T-DNA insertion mutant lines using the 35S promoter has proven to show an exceedingly low success rate for reporter detection in guard cells for unknown reasons (J.M. Kwak, G.A. Allen and I.M. Mori unpublished observation).

The *KST1 *promoter can drive reporter gene expression in guard cells and flowers in potato [24]. But the *KST1 *promoter has not been used widely in research to drive specific expression in *Arabidopsis *or other plant guard cells. In *Arabidopsis*, the *KAT1 *promoter drives primarily reporter gene expression in guard cells though the expression of the reporter was also observed in the root vascular tissue in some transgenic plants [25]. Furthermore, the *KAT1 *promoter is not sufficiently strong for high-level expression or repression in guard cells.

Here we used a guard cell specific microarray-based approach to analyze putative strong guard cell specific promoters. One candidate promoter, *pGC1 *(At1g22690), drove very strong expression of reporter genes (GUS and GFP-based calcium reporter) in guard cells of both *Arabidopsis *and tobacco. Specific gene suppression in guard cells was also achieved by *pGC1 *driving antisense repression.

## Results

### Isolation of pGC1, a strong guard cell promoter

**Figure 1:**
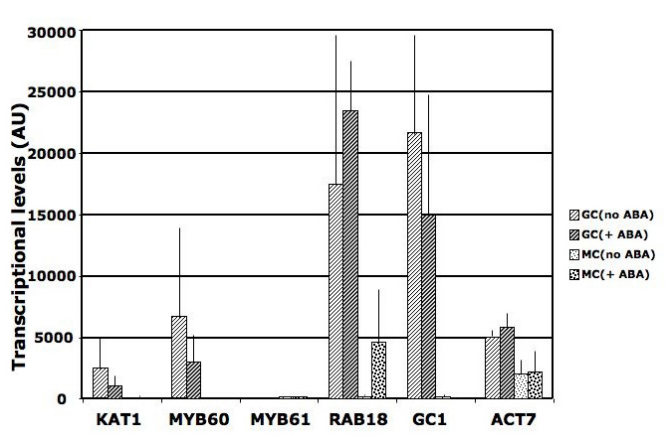
**Transcriptional profiles of guard cell expressed genes in both guard cells and mesophyll cells**. Average transcript levels of *KAT1 *(At5g46240), *AtMYB60 *(At1g08810), *AtMYB61 *(At1g09540), *RAB18 *(At5g66400), *GC1 *(At1g22690), and *AtACT7 *(At5g09810) from two independent microarrays are displayed. While *KAT1*, *AtMYB60 *and *GC1 *all exhibited guard cell-specific expression, the transcript level of *GC1 *was the highest among the three genes. *RAB18 *also exhibited very strong guard cell expression, but its expression level in mesophyll cells was strongly induced by ABA treatment.

**Figure 2:**
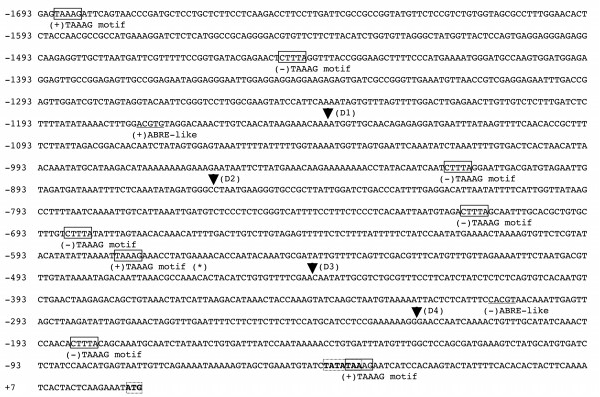
**Putative promoter sequence of *GC1***. The transcriptional start site is denoted as +1, and the putative start codon (ATG) is located at +23/+25 bp. The Dof target sites, 5'-TAAAG-3' (+) or 5'-CTTTA-3'(-), which have been shown to contribute to guard-cell specific gene expression [24], are boxed. The ABRE, abscisic acid-response element, 5'-ACGTG-3' (+) or 5'-CACGT-3' (-), are underscored and labeled. The TATA box (5'-TATATAA-3') and the start codon (ATG) are shown in bold with dotted boxes. The arrowheads mark the positions for promoter deletion analyses in Figure 4.

Guard cell-specific microarray data were analyzed side by side with mesophyll cell-specific microarray data [26] to search for strong guard cell promoter candidates with low expression levels in mesophyll cells. Additional guard cell and mesophyll cell microarray experiments were conducted covering 23,000 genes (ATH1 Affymetrix) (See Additional files 1, 2, 3, 4, 5, 6, 7 and 8). Furthermore, candidate genes were analyzed using Genevestigator to select genes with low expression levels in non-leaf tissues across more than 2000 microarray experiments [27]. Guard cells and mesophyll cells exposed to ABA were also analyzed, as ABA synthesis is induced under several stress conditions. The following criteria were used for selection of strong guard cell promoter candidates. The raw signal in guard cells was set above 10000, the raw signal in mesophyll cells was set below 1000, and the reduction or induction fold by ABA was set to be less than two. Transcriptional profiles of several genes passed these criteria (Figure 1 and Additional file 9). The putative promoters (1–2 kb upstream of the annotated ATG start codon) (Figure 2) were amplified by PCR and cloned into a GUS reporter vector. GUS staining of the T1 transgenic plants showed guard cell specific staining for one particular promoter candidate (At1g22690), designated as *pGC1*. At1g22690 is among the most highly expressed genes in guard cells. It showed relatively high expression in guard cells and low expression in mesophyll cells. At1g22690 encodes a small cysteine rich protein (119 amino acids). It belongs to the GASA family (GA-stimulated transcript (GAST1) protein homolog). A study by Wigoda et al. [28] suggested that GIP2 (a GASA protein from *Petunia hybrida*) exhibited *in planta *antioxidant activity. T-DNA insertional line in At1g22690 did not yield any noticeable stomatal phenotypes under our typical laboratory conditions (unpublished data). Furthermore, our guard cell microarray data showed that two other GASA genes also showed high expression level in guard cells (GASA 1 (At1g75750) and GASA 4 (At5g15230)).

We analyzed GC1 (At1g22690) gene expression in response to different treatments in the microarray data compiled by Genevestigator [27,29]. Among 96 treatments, 8 treatments affected At1g22690 expression more than two fold. Salt and osmotic stress dramatically deceased At1g22690 gene expression (more than 10 fold) [30]. Meanwhile, light, ABA, GA, cold or drought did not induce more than a two-fold change in gene expression of At1g22690. This suggests that *GC1 *(At1g22690) has a relatively constant expression under most common situations.

**Figure 3:**
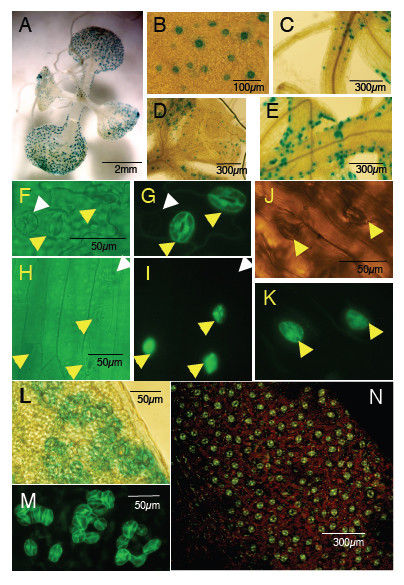
**The *GC1 *promoter mediates strong reporter expression in guard cells of wild-type *Arabidopsis *seedlings, *too many mouths *mutant and also in tobacco**. A. A two-week-old *pGC1::GUS *transgenic seedling. B. Different stages of guard cells exhibited different levels of *GUS *expression. C. Upper part of the hypocotyl. D. Young leaf and petiole. E. Leaf edge and petiole. F. & G. *pGC1::YC3.60 *was mainly expressed in mature guard cells, very weak in young or immature guard cells (white arrows in (f) & (g)). H. & I.*pGC1::YC3.60 *was expressed in guard cells on the hypocotyl. J. & K.*pGC1::YC3.60 *was expressed in guard cells on the sepal. L. & M. *pGC1 *mediated GUS (L) and GFP (M) reporter expression in clustered stomata in *too many mouths*. N. *pGC1 *mediated strong reporter gene expression in tobacco guard cells.

Interestingly, the pGC1::GUS not only delivered strong GUS expression in guard cells in leaves (Figure 3A,B), but also in guard cells in petioles and hypocotyls (Figure 3C,D,E). GUS staining from other candidate promoter-GUS fusions was either not very strong in guard cells and/or showed reporter expression in other tissues (data not shown). We therefore focused on *pGC1 *for the rest of this study. The *GC1 *promoter was also fused to a second reporter, a GFP-based calcium reporter, yellow cameleon 3.60 (YC3.60) [31]. Most T1 transgenic plants (approximately 75%) transformed with *pGC1::YC3.60 *exhibited strong guard cell specific fluorescence, indicating a high degree of guard cell expression efficiency per transformant. Some plants also showed fluorescence in some leaf epidermal cells (data not shown). However, younger or immature guard cells showed no or much less GFP expression (Figure 3F,G). Furthermore, guard cells in sepals and hypocotyls also showed GFP expression (Figure 3H,I,J,K).

We further examined whether the *GC1 *promoter could drive guard cell specific reporter expression in a guard cell development mutant, *too many mouths *(*tmm*) [32]. The *tmm *mutant was transformed with either the *pGC1::GUS *or the *pGC1::YC3.60 *construct. GUS staining showed reporter gene expression in clustered stomata (Figure 3L). Similarly, GFP expression was observed in clustered stomata in *tmm *plants transformed with *pGC1::YC3.60 *(Figure 3M).

To test if the *GC1 *promoter can drive guard cell specific reporter gene expression in plants besides *Arabidopsis*, we also transformed *pGC1::YC3.60 *into tobacco plants. Interestingly, strong guard cell GFP expression was observed in tobacco leaves (Figure 3N).

### Serial promoter deletions define a region for guard cell specificity and strength

**Figure 4:**
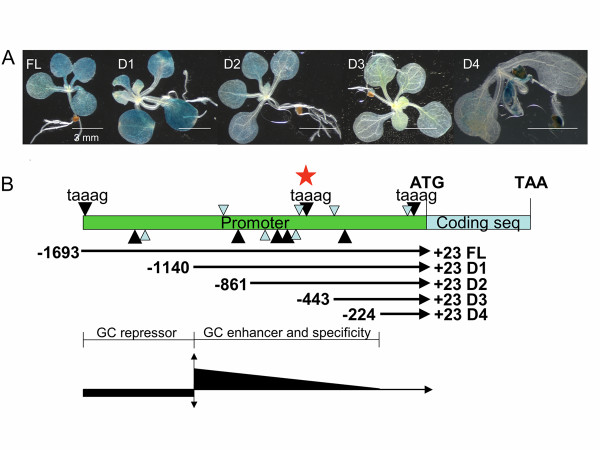
**Serial deletion of the *pGC1 *promoter defines regions for guard cell expression**. A. Representative T1 plants from different promoter::*GUS *transgenic lines. The *pGC1*(D1) (-1140/+23) promoter mediated stronger GUS expression in guard cells than the original full-length promoter (FL) (-1693/+23). GUS expression of *pGC1(D2)::GUS *and *pGC1(D3)::GUS *was weaker than that of the *pGC1(FL)::GUS *and *pGC1(D1)::GUS*. The shortest promoter *pGC1*(D4) (-224/+23) drives reporter expression in tissues and cells besides guard cells. B. Serial deletion of the *pGC1 *promoter defines regions for guard cell expression. The black arrowheads stand for TAAAG elements while the smaller gray arrowheads stand for AAAAG elements. Arrowheads on the top of the promoter line are on the sense strand while arrowheads below the promoter line are on the antisense strand. The central TAAAG on the sense strand was also marked by a star and was chosen for block mutagenesis. The region from -1693 to -1140 contains repressor elements for guard cell expression and the region from -1140 to -224 contains elements for guard cell specificity and also enhancer elements for guard cells expression.

A promoter region may contain both enhancer and repressor elements. To probe which part of the original 1716 bp promoter (full length, FL, -1693 bp/+23 bp) is required for strong guard cell specific reporter expression, four 5' truncated versions of the *GC1 *promoter were generated as D1 (-1140 bp/+23 bp), D2 (-861 bp/+23 bp), D3 (-443 bp/+23 bp), and D4 (-224 bp/+23 bp) (Figure 4). These truncated promoters were fused to the *GUS *reporter to generate the following constructs: *pGC1(D1)::GUS*, *pGC1(D2)::GUS*, *pGC1(D3)::GUS *and *pGC1(D4)::GUS*. These *GUS *reporter constructs were transformed into Columbia wild type plants side-by-side with the original *pGC1(FL)::GUS *construct. T1 seedlings (n = 50–100) from each transformation event were pooled and stained. The truncated *pGC1*(*D1*) drove similar or stronger GUS expression in seedlings than the original full-length promoter (Figure 4A), suggesting that elements in the region from -1693 bp to -1140 bp might repress promoter activity in guard cells. Promoters *pGC1*(*D2*) and *pGC1*(*D3*) led to weaker reporter gene expression in guard cells than *pGC1*(*FL*), suggesting elements in the region from -1140 bp to -443 bp might enhance the promoter activity in guard cells. The shortest promoter, *pGC1*(*D4*), drove reporter gene expression in tissues other than guard cells, such as roots and seed coats, suggesting the region from -861 bp to -224 bp was required for guard cell specific activity. This region contains 8 (T/A)AAAG elements that have been shown to be required for guard cell specific activity of the *KST1 *promoter in potato [24]. The truncated promoter, *pGC1(D1)*, showed strong guard cell expression, suggesting that it contains elements for both guard cell specificity and promoter strength.

### Calcium imaging in guard cells of intact plants

Many physiological stimuli in plant cells induce changes in the intracellular calcium concentration. Calcium acts as a secondary messenger in many signal transduction cascades [33]. Cytosolic calcium concentrations can be monitored either by chemical reporters such as the ratiometric Ca^2+^-sensitive fluorescent dye fura-2 [34,35], the genetically encoded calcium sensitive luminescent protein aequorin [14] or the fluorescent ratiometric calcium reporter yellow cameleon [12,15,36]. Stomatal closing signals, such as ABA and CO_2_, have been shown to induce calcium elevations in guard cells [16,18,19,37-42]. Spontaneous calcium transients in leaf epidermal samples have also been observed without any ABA treatment [15,43,44]. It is not clear whether spontaneous calcium transients occur in guard cells in intact plants as fura-2 injected *Vicia faba *guard cells did not show such transients [45]. A new generation calcium indicator, yellow cameleon, YC3.60, shows an enhanced calcium-dependent change in the ratio of YFP/CFP by nearly 600% compared with yellow cameleon 2.1 [31]. By combining the *GC1 *promoter with *YC3.60*, *pGC1::YC3.60*, as described before, we could observe strong guard cell expression of the *YC3.60 *in intact leaves, hypocotyls, and sepals (Figure 3).

**Figure 5:**
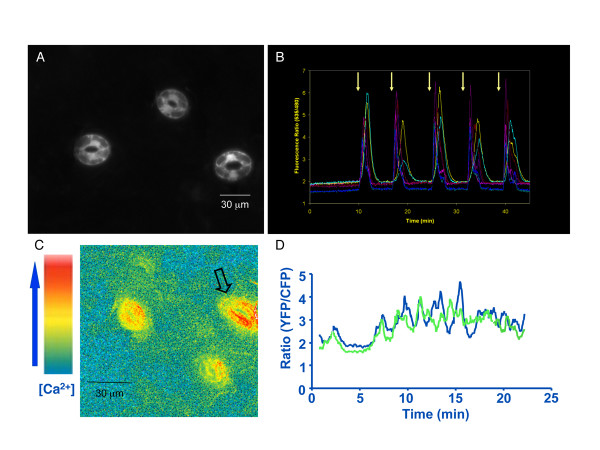
**Imposed intracellular calcium transients in *pGC1::YC3.60 *expressing guard cells and spontaneous calcium transients occur in guard cells of intact *pGC1::YC3.60 *transgenic plants**. A. Fluorescence image of leaf epidermis of *pGC1::YC3.60 *transgenic plant. Note the surrounding epidermal cells were not fluorescent. B. The 6 guard cells in panel A all produced intracellular calcium transients in response to imposed calcium oscillations. The arrows mark the switch point from the depolarizing buffer to the Ca^2+^-containing hyperpolarizing buffer (see Methods). C. A pseudo-colored ratiometric image of a leaf from an intact Col plants transformed with *pGC1::YC3.60*. The orange-yellow color indicates higher [Ca^2+^] and the blue color indicates lower [Ca^2+^]. Spontaneous calcium transients occurred in leaves of intact *Arabidopsis *plants (movies are shown as additional files 10 and 11). D. A time course (25 minutes) of the emission ratios of the two guard cells marked by an arrow in C shows that spontaneous calcium transients occur in intact *Arabidopsis *plants. The ratio was calculated for individual cells by dividing the YFP emission intensity by the CFP emission intensity.

We first measured calcium transients in intact leaf epidermis from plants transformed with *pGC1::YC3.60 *by imposing calcium oscillations as described previously [11,46]. Robust calcium transients with ratiometric changes of up to a factor of 4 relative to the baseline ratio could be observed in guard cells (Figure 5B). Ratiometric changes of approximately 0.5 were observed using *35S::YC2.1 *in response to imposed calcium transients [15,43,44,46]. This further confirmed the robust ratiometric signal to noise efficiency of YC3.60. Next, we performed calcium imaging in intact *Arabidopsis *seedlings by mounting leaves to a microscope cover glass. Two different methods were tested: the first one was to submerge only the root with water and leave the shoot in air, and the second one was to submerge the entire plant in water. Spontaneous calcium transients were detected under both conditions (Table 1). A representative calcium transients/time course is shown in Figure 5D. Interestingly, the spontaneous calcium transients of two guard cells from the same stomate were often not synchronized (Figure 5C,D and additional files 10 and 11). These experiments clearly demonstrate that spontaneous calcium transients occurred in guard cells of intact plants and were not an artifact of imaging excised epidermis and illustrate the potential of the *pGC1 *promoter as a method for driving transgene and reporter expression in guard cells.

**Table 1:** Summary of calcium imaging in guard cells of intact pGC1::YC3.60 transgenic *Arabidopsis *plants.

**Experiments**	**Plants**	**GCs analyzed**	**GCs with Spontaneous Ca2+ transients**	**Percentage %**
I	5	24	18	75
II	11	52	36	62.23
III	11	55	36	65.45
IV	9	54	24	44.44
				
Total	**36**	**185**	**114**	61.78%

Only roots were submerged in water in experiment I. Both leaves and roots were submerged in water in experiments II, III, and IV.

### The use of *pGC1 *to manipulate specific gene expression in guard cells

**Figure 6:**
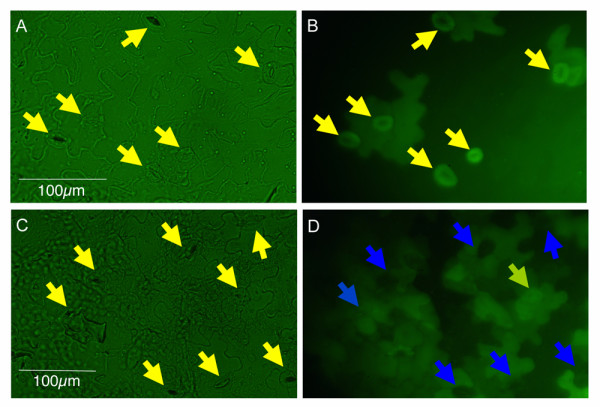
***pGC1(D1)::anti-GFP *caused reduction of *GFP *expression in guard cells of *35S::GFP *plants**. A. Leaf epidermis of a *35S::GFP *transgenic plant (bright field with GFP filter). The arrows mark stomata. B. The fluorescence imaging of same leaf epidermis shown in A. Stomata are marked by yellow arrows. Note that both the guard cells and surrounding epidermal cells are fluorescent. C. Leaf epidermis of a T1 transgenic plant expressing *pGC1(D1)::anti-GFP *in the *35S::GFP *background. All stomata are marked by yellow arrows. D. The fluorescence imaging of the same leaf epidermis shown in C. Note that 7 (marked by blue arrows) out of 8 stomata showed reduced GFP expression compared with the surrounding epidermal cells. One pair of guard cells (marked by the yellow arrow) still exhibited moderate GFP expression. This stomate was relatively immature compared with the other 7 stomata.

Manipulation of specific gene expression in guard cells, either by highly expressing the wild-type gene or a dominant mutant form, or reducing its expression in guard cells, would be very powerful to probe a specific gene function in guard cells. To further explore the application of the *GC1 *promoter, we took the antisense approach to analyze reduction of gene expression in guard cells. For this purpose, a *35S::GFP *transgenic line with stable GFP expression in both guard cells and epidermal cells (Figure 6A,B) was transformed with a *pGC1(D1)::anti-GFP *construct (*anti-GFP *fused to the truncated *GC1 *promoter *pGC1(D1)*). 34 out of 40 T1 plants of *35S::GFP *plants transformed with *pGC1(D1)::anti-GFP *showed greatly reduced GFP expression in guard cells while the GFP expression level in epidermal cells was unchanged (Figure 6C,D). These observations suggest a remarkable antisense repression efficiency using *pGC1(D1)*. Interestingly, less suppression of GFP expression was observed in immature guard cells (yellow arrow in Figure 6D). This is consistent with the observation that *pGC1 *drove less reporter gene expression in immature guard cells (Figure 3G). This experiment strongly indicates that an antisense approach can be used to reduce expression of selected genes in guard cells without affecting its expression in other cell types.

## Discussion

We report the identification of a strong *Arabidopsis *guard cell promoter, *pGC1*. Promoter::reporter fusion analyses showed strong guard cell specific reporter gene expression in wild-type *Arabidopsis *plants and the guard cell development mutant, *too many mouths *[32] and also tobacco plants. Serial deletions of the *GC1 *promoter defined regions for guard cell expression. Calcium imaging in guard cells in intact plants was made possible via the combination of the *GC1 *promoter and a new generation of calcium reporter, *YC3.60 *[31]. The *GC1 *promoter was also powerful for knocking down specific gene expression in guard cells using an antisense approach.

### Comparison between the *GC1 *promoter and other known guard cell promoter

As the central regulator of water transpiration and CO_2 _uptake, guard cells have been developed as an integrative model system to investigate interplay among ion channel/transporter activities, light, plant hormones, secondary messengers, the cytoskeleton and membrane trafficking in regulating the physiological output: the stomatal aperture [2,4,5,47,48]. Several guard cell promoters have been reported. The *KAT1*(At5g46240) promoter delivered specific reporter expression in guard cells even though it sometimes induced reporter expression in other cells and tissues such as roots and inflorescences [25]. *AtMYB60 *(At1g08810) also showed specific expression in guard cells based on promoter::GUS and promoter::GFP study [49]. *AtMYB61*(At1g09540) has also been shown to be mainly expressed in guard cells [50]. Based on our guard cell-specific microarray data, we estimated the average transcription levels in Figure 1 and additional file 9. The *AtMYB61 *gene expression signal was the lowest among these genes. In the case of *KAT1*, its expression in guard cells was much higher than that in mesophyll cells. But its raw signal was approximately 5 to 10 fold lower than that of *GC1*. *AtMYB6*0 also exhibited highly guard cell specific expression compared with its expression in mesophyll cells. However, the raw signal of *AtMYB60 *was only approximately one third of that of *GC1*. Furthermore, *AtMYB60 *is also highly expressed in seeds based on Genevestigator microarray analyses [27,29,51-54]. Similarly, *RAB18 *(At5g66400) is also highly expressed in seeds besides its strong expression in guard cells. *pGC1 *drove very strong and specific reporter gene expression in guard cells (expression is very low in non-leaf tissues/organs), although reporter gene expression was observed in epidermal cells in some plants transformed with the *pGC1::YC3.60 *(data not shown). In summary, the *GC1 *promoter is a very strong guard cell promoter among those analyzed.

### Spontaneous calcium transients in guard cells

Our current study with intact *Arabidopsis *plants using the genetically encoded calcium reporter YC3.60 driven by the *GC1 *promoter showed that spontaneous calcium transients occurred in guard cells in intact *Arabidopsis *plants. This is consistent with previous observations of spontaneous calcium transients in *Arabidopsis *guard cells [15,43,44]. However, the mechanisms causing spontaneous calcium transients are not yet characterized in depth. Several lines of evidence suggest a connection between hyperpolarization of the guard cell plasma membrane and spontaneous calcium transients in guard cells. In experiments where membrane potential and [Ca^2+^]_cyt _were measured simultaneously, hyperpolarization caused ABA-induced [Ca^2+^]_cyt _increases. Maintaining guard cells in a more hyperpolarized state produced spontaneous [Ca^2+^]_cyt _oscillations in *Vicia faba *guard cells [38], in a sub-population of *Commelina *guard cells [39] and in *Arabidopsis *guard cells [43]. Calcium imaging analyses in intact *Arabidopsis *plants using pGC1::YC3.60 show that spontaneous calcium transients also occur in intact plants. These spontaneous Ca^2+ ^transients may also be the result of integrated signaling by multiple stimuli converging in guard cells, such as light conditions, CO_2 _and water balance. In *Vicia faba *no spontaneous calcium transients were observed in guard cells in intact plants [45]. In this case fura-2 (ca. 100 μM) was injected into guard cells. High concentrations of fura-2 may inhibit spontaneous calcium elevations, as loading the close fura-2 analogue, BAPTA, into *Arabidopsis *guard cells effectively inhibits these calcium transients [44]. By contrast, the estimated yellow cameleon concentration in guard cells of pGC1::YC3.60 transgenic plants was approximately 1 μM (see Methods). The lower concentration of yellow cameleon should interfere less with guard cell calcium homeostasis and could monitor more faithfully calcium concentration dynamics. Note that low concentrations of injected fura-2 also allowed resolution of repetitive calcium transients in guard cells [38,39]. Note that BAPTA-derived fluorescent dyes such as fura-2 and indo-1 have certain complementary advantages to cameleon, as they can be loaded into cells that are not easily transformed [55] and these dyes can report rapid millisecond scale Ca^2+ ^transients that occur in neurons[56], but have presently not yet been reported in plants using fura-2 or indo-1.

Circadian calcium oscillations at the whole plant leaf level with a daily rhythm have been demonstrated by several groups using aequorin as the calcium reporter [57-59]. Most likely this circadian calcium oscillation results from synchronous changes in baseline cytosolic calcium in a cell population [60]. As the circadian calcium oscillation is related to the baseline of intracellular calcium, the rapid spontaneous calcium transients in individual guard cells likely would be filtered from circadian calcium measurements [60]. Repetitive calcium transients may reflect functions that include continuous calcium homeostasis between extracellular calcium, cytoplasmic calcium, and intracellular calcium stores. Spontaneous calcium transients in guard cells also correlate with the recent proposed calcium sensor priming hypothesis for calcium specificity in signaling, in which the stomatal closing signals ABA and CO_2 _are proposed to prime (de-inactivate) calcium sensitive steps that mediate stomatal closing [44,61].

### (T/A)AAAG cis elements and guard cell specific expression

(T/A)AAAG, a binding motif for Dof zinc finger transcription factors, has been suggested to play a critical role for guard-cell specific expression of *KST1 *promoter activity in potato based on block mutagenesis [24]. However, the putative promoter regions (1800 bp before ATG start codon) for *AtACT7 *(At5g09810), *KAT1 *(At5g46240), *RAB18 *(At5g66400), *AtMYB60 *(At1g08810), *AtMYB61 *(At1g09540) and *GC1 (At1g22690) *all contain a similar number of Dof factor binding motifs, the (T/A)AAAG elements, even though some of them do not show guard cell expression preference (see additional file 12). *AtMYB61*, which showed low expression in guard cells (Figure 1), contains 29 (T/A)AAAG elements in its putative promoter region, while the *AtACT7 *promoter contains 23 (T/A)AAAG elements. Promoter truncation suggests that the region from -861bp to -224 bp in the *GC1 *promoter contains elements for guard cell specific promoter activity (Figure 4). This region contains 8 (T/A)AAAG elements. However, block mutagenesis of the central TAAAG motif on the sense strand (marked by a star in Figure 4B) in this region did not affect reporter expression in guard cells (data not shown). Thus the (T/A)AAAG element alone may not explain why *GC1 *and other guard cell-specific genes exhibited guard cell-specific expression.

## Conclusion

In this report, we pursued microarray (ATH1) analyses of guard cell expressed genes and used the information to isolate and characterize a strong guard cell promoter, *pGC1*. We analyzed the potential of *pGC1 *as a tool for manipulating gene expression in guard cells. The *GC1 *promoter was used to test several experimental manipulations. The *GC1 *promoter was used to express the calcium reporter YC3.60 in guard cells. This enabled us to perform calcium imaging experiments in guard cells of intact *Arabidopsis *plants. Our previous research has shown that for T-DNA insertional mutants, hundreds of transformants often needed to be generated to obtain at best a few lines expressing a reporter gene in guard cells when using the 35S promoter. Use of the *GC1 *promoter provides a method to dramatically increase the success rate of reporter gene expression. Furthermore, guard cell-specific antisense GFP expression using the *GC1 *promoter efficiently silenced GFP expression in guard cells of 35S::GFP transgenic plants. These data and the high transformation efficiency together suggest that the *GC1 *promoter provides a powerful tool for manipulating the expression of guard cell signaling components and for expressing reporters of diverse secondary messengers. Thus the *GC1 *promoter provides a method to enhance monitoring of signaling events in guard cells in response to different treatments and to study whole plant responses in guard cell specific transgenic mutants.

## Materials and Methods

### Plant material

*Arabidopsis thaliana *(Columbia ecotype) plants were used for transformation experiments unless otherwise specified. The 35S::GFP transgenic line was generated for a previous study [62]. The guard cell development mutant, *too many mouths*, was a kind gift from Dr. Fred Sack at the University of British Columbia, Vancouver.

### GeneChip microarray experiments

Plant growth, ABA treatment, guard cell protoplast isolation, and RNA extraction were performed as previously described [26]. Affymetrix *Arabidopsis *ATH1 genome arrays (Santa Clara, CA) were used, representing approximately 23,000 genes. Transcripts were amplified, labeled, and hybridized at the University of California, San Diego Gene Chip Core facility. For each condition (with or without ABA treatment, guard cell or mesophyll cell), two independent hybridizations were performed. Transcriptional inhibitors (33 mg/L actinomycin D and 100 mg/L cordycepin) were added during protoplast isolation for RNA samples for four chip hybridizations as described [26]. ATH1 microarray data were deposited at MIAMExpress [63] with an accession number E-MEXP-1443 and also on our laboratory's website for public downloading (see Additional files 1, 2, 3, 4, 5, 6, 7 and 8).

### Construction of recombinant plasmids

To amplify the *GC1 *(At1g22690) promoter from the Col genomic DNA by PCR, primers YZ27 (5'-CATGCCATGGatttcttgagtagtgattttgaag-3', right before the ATG start codon with NcoI site) and YZ28 (5'-ACGCGTCGACgagtaaagattcagtaacccg-3', 1693 bp upstream of the transcriptional start (Figure 2) with SalI site) were utilized. The PCR product was cloned into pGEM-Teasy vector (Invitrogen, Carlsbad, CA) to create **pGEM-T-pGC1**.

To clone the *GC1 *promoter into the pBI101 vector, pGEM-T-pGC1 was first cut by NcoI. The sticky end was then filled-in by T4 DNA polymerase (New England BioLabs) to create a blunt end. The pGC1 fragment was then released by SalI digestion. Meanwhile, the destination vector, pBI101, was cut sequentially by SmaI and SalI. The pGC1 fragment was then inserted upstream of the *GUS *reporter gene in the pBI101 vector to create pBI101-**pGC1::GUS **construct (simplified as **pGC1::GUS**).

To create the 5'-deletion series of the *pGC1 *promoter, primer YZ27 was used with primers YZ159 (5'-GCGTCGACatggttgcaacagagaggatga-3', 1141 bp upstream of the transcriptional start, D1), YZ160 (5'-GCGTCGACctaatgaagggtgccgcttattg-3', 861 bp upstream of the transcriptional start, D2), YZ161 (5'-GCGTCGACcaatattgcgtctgcgtttcct-3', 466 bp upstream of the transcriptional start, D3) and YZ162 (5'-GCGTCGACgaaccaatcaaaactgtttgcata-3', 224 bp upstream of the transcriptional start, D4) respectively for genomic PCR to amplify *pGC1(D1)*, *pGC1(D2)*, *pGC1(D3) *and *pGC1(D4) *respectively (Figure 4). The PCR fragments were then cloned into *pGEM-T-easy *vector and then subcloned into *pBI101 *vector to create *pBI101*-***pGC1(D1)::GUS***, *pBI101*-***pGC1(D2)::GUS***, *pBI101*-***pGC1(D3)::GUS***, and *pBI101*-***pGC1(D4)::GUS***.

To create *pBI101*-***pGC1::YC3.60 ***construct, *YC3.60 *was first released from *pcDNA3-YC3.60 *[31] by EcoRI/BamHI double digestion. Then the BamHI-5'-*YC3.60*-3'-EcoRI fragment was cloned into *pSK *vector (prepared by EcoRI and BamHI digestion) to create *pSK-YC3.60 *construct. The *pSK-YC3.60 *was then digested with NotI and NcoI to receive NotI-5'-*pGC1*-3'-NcoI fragment from *pGEM-T-pGC1*. This ligation resulted in the *pSK-pGC1::YC3.60*. The *pGC1::YC3.60 *fragment was released by SalI/SacI double digestion, meanwhile the *pBI101 *vector was digested with SalI/SacI to remove the *GUS *reporter gene. The *pBI101*(SalI/SacI) was ligated with SalI-5'-pGC1::YC3.60-3'-SacI to create *pBI101*-***pGC1::YC3.60 ***construct.

To create *pGreenII 0179*-***pGC1(D1)::anti-GFP ***binary vector with hygromycin selective marker in plant, the *35S *terminator was amplified with YZ439 (5'-AAGAGATCTATCTAGAGTCCGCAA-3', with XbaI) and YZ440 (5'-GCACGCTCGAGCTCgtcactggattttggttttagg-3', with SacI site) from vector pAVA319 [64]. The PCR product was then subsequently digested with XbaI and SacI. The 5'-XbaI-35S terminator-SacI-3' was ligated into *pGreenII 0179*-XabI...SacI to create *pGreenII 0179-terminator*. The *pGC1(D1) *was released from *pGEM-T-pGC1(D1) *by NotI digestion, then filled-in, then cut by SalI to create 5'-SalI-pGC1(D1)-NotI(filled-in blunt end). Meanwhile, the pGreenII 0179-terminator was doubled digested with SalI and EcoRV. These two fragments were ligated to generate *pGreenII 0179-pGCP(D1)-terminator *vector. The antisense *GFP *was amplified with primers YZ449 (5'-ACATGCCATGGttacttgtacagctcgtccatgcc-3', reverse end of GFP with NcoI) and YZ513 (5'-ctagTCTAGA**atg**gtgagcaagggcgagg-3', start of GFP with XbaI). The PCR fragment was double digested with NcoI and XbaI. The *pGreenII 0179-pGC1(D1)-Terminator *was also double digested with NcoI and XbaI. The pGeenII 0179-pGC1(D1)-Terminator fragment was ligated with 5'-NcoI *-anti-GFP*-XbaI-3' to produce *pGeenII 0179*-***pGC1(D1)::anti-GFP ***binary construct.

The central TAAAG motif (-579-->-575) on the sense stand was changed to CGGGA by block mutagenesis using the QuickChange Site-Directed Mutagenesis Kit from Stratagene (La Jolla, California).

### Arabidopsis transformation and selection

The binary constructs, *pBI101*-***pGC1::YC3.60***, *pBI101*-***pGC1::GUS***, *pBI101*-***pGC1(D1)::GUS***, *pBI101*-***pGC1(D2)::GUS***, *pBI101*-***pGC1(D3)::GUS ***and *pBI101*-***pGC1(D4)::GUS ***were transformed into the *Agrobacterium tumefaciens *strain GV3101 by electroporation. The transformants were selected on LB plates with both kanamycin (selective marker for the construct) and gentamycin (selective marker for the Agrobacterium). *Arabidopsis *plants were then transformed by *Agrobacterium* GV3101 hosting respective constructs following the dipping method as described by Clough and Bent [65]. The T0 seeds were selected on 1/2 MS plates with 50 μg/ml kanamycin.

In the case of *pGreenII 0179*-***pGC1(D1)::anti-GFP***, the GV3101 with the helper plasmid *pSOUP *was used as the host strain, and the selection for *Agrobacterium* transformants was carried on LB plates with Kanamycin, gentamycin, and tetracyclin. This was used to transform 35S::GFP transgenic plants (kanamycin resistant). The T0 seeds were selected on 1/2 MS plates with 25 μg/ml hygromycin (Roche).

### GUS staining

Seedlings were stained following a previously described protocol [62].

### Epi-fluorescence image acquisition

Transgenic *Arabidopsis *seedlings or sepals of *pBI101-pGC1::YC3.60 *were simply placed between a microscope slide and a cover glass. A Nikon digital camera was attached to the microscope. Exposure time for the bright image is 5 seconds and 15–25 seconds for fluorescence image (excitation wavelength is 440 nm). For 35S::GFP plants and 35S::GFP plants transformed with pGreenII 0179-*pGC1(D1)::anti-GFP*, intact leaf epidermis were used for epi-fluorescence image acquisition.

### Tobacco plant transformation

*In vitro *sterile shoot cultures of *Nicotiana tabacum *cv. SR1 were maintained on 1/2 MS agar medium containing 15 g/l sucrose. The pH was adjusted to 5.5 before autoclaving. The tobacco culture was grown at 25°C, with a light/dark cycle of 16/8 h (light intensity was approximately 70 μmol m^-2 ^s^-1^). Stable transformation of *Nicotiana tabacum *SR1 with *pBI101*-***pGC1-YC3.60 ***was performed as described previously [66]. Transgenic regenerated tobacco shoots were selected by kanamycin (100 μg/ml) resistance and were then transferred on 1/2 MS agar medium containing 15 g/l sucrose supplemented with kanamycin (100 μg/ml) and cefotaxime (200 μg/ml). T1 regenerated plants, which were able to set up root organogenesis in presence of kanamycin, were then analyzed for cameleon expression.

### Confocal analysis of transgenic tobacco

The tobacco leaves of plant transformed with *pBI101*-***pGC1-YC3.60 ***were observed with a Leica TCS SP2 laser confocal microscope (Leica Microsystems). For cameleon detection, excitation was at 514 nm and emission between 525 and 540 nm. The images acquired from the confocal microscope were processed using Image J [67].

### Calcium imaging and imposed Ca^2+ ^Transients

All calcium imaging in this work was performed with a TE300 inverted microscope using a TE-FM Epi-Fluorescence attachment (Nikon Inc. Melville, NY). Excitation from a 75 W Xenon lamp (Osram, Germany) was always attenuated 97% by using both 4× and 8× neutral density filters (3% transmission) to reduce bleaching of reporters during time-resolved imaging. Wavelength specificity was obtained with a cameleon filter set (440/20 excitation, 485/40 emission1, 535/30 emission2, 455DCLP dichroic; filter set 71007a Chroma Technology, Rockingham, VT). Filter wheel, shutter and CoolSNAP CCD camera from Photomerics (Roper Scientific, Germany) were controlled with Metafluor software (MDS, Inc., Toronto, Canada).

Intact leaf epidermes of *pGC1::YC3.60 *transgenic plants were prepared for microscopy as described in Mori et al. (2006) [11]. On the microscope, intact epidermis was perfused with depolarization buffer (10 mM MES-Tris buffer, pH 6.1 containing 25 mM dipotassium imminodiacetate, and 100 μM BAPTA) for 10 minutes to obtain a background. Subsequently hyperpolarizing buffer containing Ca^2+ ^(10 mM MES-Tris buffer, pH 6.1, 1 mM dipotassium imminodiacetate, and 1 mM CaCl_2_) was applied for 2 minutes intervals, followed by 5 minutes of depolarizing buffer.

### Calcium imaging in guard cells of intact plants

Both intact leaves and intact plants were used in this study. Medical adhesive (Hollister Inc., Libertyville, IL) was used to attach leaves to microscope cover glasses. A paintbrush was used to gently press the leaf to the coverslip. In the case of intact plants two different methods were followed. The first method was to submerge only the root with water while the shoot was left in air. The second method was to completely submerge entire seedlings in water. Sometimes submerging only the root but not the shoot caused the leaf attached to the cover slip to show wilting in less than 10 minutes with subsequent closure of the stomata. Most of the intact plant imaging experiments were therefore carried out by submerging both the shoot (leaves) and the root in water. The submersion of the entire plant prevented the leaf from drying out and no stomatal closure was observed for more than 50 minutes. The imaging protocol was the same as in Mori et al., 2006 [11].

### Estimation of yellow cameleon concentration in guard cells

Recombinant yellow cameleon protein was isolated after expression in E coli. Recombinant cameleon protein was then added at defined concentrations to a glass cover slip for fluorescence imaging. Then two additional cover slips were used to create a slanted gradient of cameleon solution thicknesses. This enabled analysis of various solution thicknesses in the range of stomatal guard cell thicknesses. Diluted yellow cameleon protein solutions at different concentrations were analyzed and the florescence intensity was measured for each concentration at various thicknesses. Calibration curves were generated for protein concentrations and florescent intensities at different thicknesses. This was utilized to estimate the yellow cameleon protein concentration in guard cells of pGC1::YC3.6 transgenic plants.

## Competing interests

The guard cell specific and mesophyll cell specific microarray data generated in this study will be available at public data bases [63] and the *GC1 *promoter construct described in this manuscript will be made freely available to the academic and non-profit laboratories and also to PIPRA-associated projects. The authors' institution has submitted a patent application that includes the *GC1 *promoter.

## Authors' contributions

YY performed the second sets of guard cell/mesophyll cell microarray experiments, isolation of the *GC1 *promoter, construction of all plasmids, transformation of *Arabidopsis *plants, analyses of GUS and yellow cameleon expression patterns and wrote the manuscript. AC did calcium imaging in intact *Arabidopsis *plants and transformation of tobacco plants. NL did the first sets of guard cell/mesophyll cell ATH1 microarray experiments. RS did the imposed calcium oscillation and analysis of the yellow cameleon concentration in guard cells. JIS proposed the study, participated in its design and coordination and co-wrote the manuscript with YY. All authors read and approved the final manuscript.

## Supplementary Material

Additional file 1 Experiment I-Guard cells without ABA (JS33.xls). Actinomycin and Cordycepin were added during protoplast isolation. Click here for file

Additional file 2 Experiment I-Guard cells treated with 100 μM ABA (JS34.xls). Actinomycin and Cordycepin were added during protoplast isolation. Click here for file

Additional file 3 Experiment I-Mesophyll cells without ABA (JS35.xls). Actinomycin and Cordycepin were added during protoplast isolation. Click here for file

Additional file 4 Experiment I-Mesophyll cells treated with 100 μM ABA (JS36.xls). Actinomycin and Cordycepin were added during protoplast isolation. Click here for file

Additional file 5 Experiment II-Guard cells without ABA (JS85.xls). Click here for file

Additional file 6 Experiment II-Guard cells treated with 100 μM ABA (JS86.xls) Click here for file

Additional file 7 Experiment II-Mesophyll cells without ABA (JS87.xls). Click here for file

Additional file 8 Experiment II-Mesophyll cells treated with 100 μM ABA (JS88.xls). Click here for file

Additional file 9 Guard cell promoter candidate gene expression in GC and MC. Click here for file

Additional file 10 Representative movie 1 of spontaneous calcium transients occurring in guard cells of intact *pGC1::YC3.60 *transgenic plants. The time compression factor is 30 times. Click here for file

Additional file 11 Representative movie 2 of spontaneous calcium transients occurring in guard cells of intact *pGC1::YC3.60 *transgenic plants. The time compression factor is 30 times. Click here for file

Additional file 12 Multiple (T/A)AAAG elements are present in all the examined promoters. Click here for file
