# Supplementary material for: Isolation of a strong Arabidopsis guard cell promoter and its potential as a research tool
Source: Plant Methods. 2008 Feb 19;4:6. doi: 10.1186/1746-4811-4-6 (PMC2323621; doi:10.1186/1746-4811-4-6)
Supplement: Additional file 9 — Guard cell promoter candidate gene expression in GC and MC. [file 1746-4811-4-6-S9.doc]

**Additional File 3.** Guard cell promoter candidate gene expression in GC and MC.
