# Supplementary material for: Isolation of a strong Arabidopsis guard cell promoter and its potential as a research tool
Source: Plant Methods. 2008 Feb 19;4:6. doi: 10.1186/1746-4811-4-6 (PMC2323621; doi:10.1186/1746-4811-4-6)
Supplement: Additional file 12 — Multiple (T/A)AAAG elements are present in all the examined promoters. [file 1746-4811-4-6-S12.doc]

**Additional file 4**. Multiple (T/A)AAAG elements are present in all the examined promoters.

S: sense strand. AS: antisense strand.
